# Editorial: Microbial Stress: From Sensing to Intracellular and Population Responses

**DOI:** 10.3389/fmicb.2020.01667

**Published:** 2020-07-31

**Authors:** Daniela De Biase, John P. Morrissey, Conor P. O'Byrne

**Affiliations:** ^1^Department of Medico-Surgical Sciences and Biotechnologies, Laboratory Affiliated to the Istituto Pasteur Italia – Fondazione Cenci Bolognetti, Sapienza University of Rome, Latina, Italy; ^2^School of Microbiology, Centre for Synthetic Biology and Biotechnology, Environmental Research Institute, APC Microbiome Institute, University College Cork, Cork, Ireland; ^3^Microbiology, School of Natural Sciences, College of Science, National University of Ireland, Galway, Ireland

**Keywords:** acid stress, second messengers, transcriptional regulation, cyanobacteria, pathogens, organic acids, oxidative stress, biotechnology

We initially devised this Research Topic (RT) as a valuable initiative to collect high-quality scientific articles from the participants of the 4th European Federation of Biotechnology (EFB) Microbial Stress meeting held in Kinsale, Ireland, April 2018. The scope of the RT is based on the scientific content of that “*Microbial Stress: from Systems to Molecules and bac*k” meeting. Indeed, over 40% of the articles eventually accepted for publication were contributed by meeting participants, but notably the remaining 60% was contributed by authors that work in this field. The collection of 22 original research and 2 review articles, contributed by 163 authors collectively, deal with the many different aspects of the microbial responses to biotic and abiotic stresses, relevant to many fields: from host-pathogen interactions to biotechnology, from bioremediation to food processing, from molecular and single-cell to population studies. The RT showcases the rapid developments of the microbial stress research on a range of microorganisms and stress conditions, and confirms that understanding microbial physiology under stress can be a trigger for the development of new methodologies as well as helping to integrate the knowledge from many different microbiological fields of research.

The retrospective analysis of the articles contributed to this RT allowed them to be assigned to one of four main sub-topics: (i) impact of weak organic acids and low pH on micro-organisms, from clinical to biotechnological contexts; (ii) adaptive responses in microbial pathogens to abiotic/environmental stress; (iii) oxidative and metal stress, from clinical to bioremediation contexts, and (iv) regulation of transcription and translation under stress, from epigenetic aspects to the role of second messengers and sRNAs.

## Weak Organic Acids and Low pH Stress

An area of intensive research deals with the microbial responses to acids (inorganic or organic) because these are common encounters that can affect or even be a threat to microbial growth, with implications in many area of clinical and applied microbiology research.

Using a bioinformatic analysis, Pennacchietti et al. showed that the glutamine-dependent acid resistance (AR2_Q) system in enteric bacteria is often associated with the glutamate-dependent AR system, the most potent AR system reported to date (De Biase and Pennacchietti, 2012). In addition, they developed a simple to perform, fast, and sensitive colorimetric assay which allowed the detection of the acid glutaminase, a key enzyme in AR2Q. The assay can find useful application in high-throughput phenotypic screens.

The use of weak organic acids was covered by three studies. Bushell et al. described a comprehensive characterization of the growth response of *Pseudomonas aeruginosa* to several organic acids over a range of pH values, with the long-term goal of using them in the treatment of topical infections caused by this pathogen. In their study, they found that the effects on the organic acids on growth could be best modeled with a non-parametric Gaussian process regression (rather than a parametric logistic approach), a finding that will undoubtedly help with modeling the growth behavior of other pathogens. Another study by Lourenço et al. provides insight into the effect of pH 4.0 and acetic acid or lactic acid on the efficacy of different azoles employed to treat vaginal candidiasis caused by two pathogenic fungi *Candida albicans* and *Candida glabrata*. In particular, acetic acid was shown to improve significantly the efficacy of all azoles tested, even on strains that were azole resistant. Thus, both studies point to an important role of these acids to treat infections or improve the efficacy of current therapeutic treatments.

A further article on yeast by Watcharawipas et al. reviewed the *Saccharomyces cerevisiae* response to sodium acetate. Sodium and acetic acid independently cause stress and activate appropriate responses in yeast but this article addressed the combined response to both. This illustrated the involvement of well-studied stress response mechanisms such as the Rim101 and Hog1 pathways, as well as a role for the ubiquitin ligase Rsp5. Rim101 is a transcriptional activator, mainly studied for its role in adaptation to alkaline stress, whereas Hog1 is a protein kinase that is a key mediator of the response to osmotic shock. It is still unknown how the different pathways interact to deliver an integrated response to combined stressors with different modes of action.

A link between acid stress and osmotic stress was studied by Chakraborty and Kenney who investigated the role of OmpR in *Salmonella enterica* serovar Typhimurium and *Escherichia coli* strain MG1655. The authors demonstrated that the acid stress regulon is rather different in the number of affected genes between the two microorganisms, whereas the number of genes affected under osmotic stress is similar. Notably, in both microorganisms, OmpR repressed *gltA*, coding for citrate synthase, which otherwise negatively affected cell growth during stress. Moreover, OmpR binding affinity for DNA increased at acidic pH. Control of intracellular enzyme activities in response to acid frequently occurs via conformational changes (Gut et al., 2006) and these mechanisms contribute significantly to the physiological response of the cell to acid stress, even when changes at the expression level are not detected.

## Microbial Pathogens Adaptive Responses to Stress

Using a label-free relative quantitative proteomics approach, Zai et al. performed a detailed study aimed at dissecting the responses of *Brucella abortus* to different stresses singly and when applied all together, the most likely situation encountered by this intracellular facultative microorganism when infecting the host. Overall the results using the multi-stress condition suggested that *B. abortus* by decreasing the oxidation of nutrients and amino acid use, reducing the secondary metabolite biosynthesis, enhancing iron acquisition and two-component systems better adapted to the intracellular environment. The importance of stress adaptation in the human food-borne pathogen *Listeria monocytogenes* is highlighted in a broad review by  Bucur et al. The regulatory mechanisms that underpin thermal adaptation, acid resistance, osmoregulation, and other food processing/preservation stresses were all comprehensively reviewed. The adaptability of this pathogen makes it a significant challenge for food producers, and remains a significant public health risk.

Some pathogens, such as *Yersinia ruckeri*, have optimal growth at 28°C, but cause outbreaks in the fish host at 18°C. To gain insights into the genes preferentially expressed at lower temperature and which could play a role in virulence,  Mendez et al. used Mini-*Tn5-lux-lac Km2* transposon to generate a library of 14,724 *Y. ruckeri* transconjugants, out of which 168 clones displayed β-galactosidase activity higher at 18°C than at 28°C. The *acrR* and *osmY* genes were analyzed in further detail by *in vivo* and *ex vivo* analysis of their promoters activation in different fish tissue during the colonization process via bioluminescence. The latter represents an interesting approach to significantly reduce the number of fish used in this kind of experiments. Another *in vivo* model, *Galleria mellonella* larvae, was used by Lee et al. to demonstrate that YfdX deficiency enhanced *S. enterica* serovar Typhi virulence while decreasing its susceptibility to penicillin G and carbenicillin. Through a combination of structural analyses (SEC-MALS, SAXS, crystallography) and mutagenesis studies the authors demonstrated that the tetrameric enzyme undergoes dissociation into dimers when the pH is increased from 5.5 to 8.0. Unlike the monomer, the oligomers were responsible for YfdX effectiveness *in vivo*, though the authors do not known if at different level/extent.

*Metarizhium acridum* alcohol dehydrogenase (*Ma*ADH1) expression and deletion effects on the growth and sporulation of this entomopathogenic fungi were investigated by Zhang et al. *M. acridum* belongs to a group of promising agents for biological control of pest insects such as locust and grasshopper in Africa, Asia and Australia. The authors showed that *Ma*ADH1 supports fungal growth and sporulation very likely because the enzyme detoxifies from acetaldehyde, in particular under hypoxic conditions.

A detailed analysis (hourly) of the transcriptome profile during the *E. coli* growth cycle in LB medium by Smith et al. revealed that the KEGG pathways “Ribosome” and “Microbial metabolism in diverse environments” were the most overrepresented in the different phases of growth (lag, exponential and stationary) when pH and nutrient availability change. Moreover, by using the persister phenotype as a proxy for changes in populations-wide heterogeneity, persisters were shown to increase during growth and the response of persisters formation to antibiotics was not only growth phase-dependent, but also affected by the composition of the medium containing the antibiotic. Thus, medium composition is an important consideration when screening for antibiotics against persisters.

## Oxidative and Metal Stress

An interesting observation was made by Vijay et al. about the ultrastructure of *Mycobacterium tuberculosis* from sputum and from clinical isolates exposed to oxidative stress, antibiotic isoniazid or iron deprivation *in vitro*. Using Transmission Electron Microscopy, the authors demonstrated a significant reduction in thickness of the triple-layered cell envelope and the accumulation of intracytoplasmic lipid inclusions in all isolates exposed to the above stresses, but not in the *M. tuberculosis* H37Rv reference laboratory strain. The cellular adaptations in clinical isolates may well represent a signature of dormancy and antibiotic tolerance, making them possible targets for antibiotic treatment.

Oxidative stress is also encountered by pathogenic Group B *Streptococcus* in macrophages when they colonize the human female host. Korir et al. investigated the role of a putative NADH peroxidase Npx in protecting against phagosome-associated oxidative stress. They showed that a mutant lacking the corresponding gene (*npx*) was compromised for H_2_O_2_ survival and growth in human macrophages.

In yeast, oxidative stress is an issue both in pathogenic and biotechnological settings. For biotechnology, it is of interest to find ways to improve oxidative stress resistance and two papers addressed this. First, Vázquez et al. showed the protective effects of melatonin in different yeasts used in the beverage industry. This molecule is naturally produced by yeasts and knowledge of its protective effect open up possibilities to generate overproducing strains with enhanced stress tolerance. Liu et al. took a completely different approach that focused on creating a glutathione-dependent disulfide oxidoreductase gene (*GRX1*) with a noisy promoter. This also led to increased resistance to oxidative stress and highlights some interesting points around stochasticity and population structure: for example, whether natural variability is a positive or negative feature when engineering strains for biotechnology. This idea that yeast cultures display population heterogeneity is the focus of a separate study on the biotechnological yeast *Pichia pastoris* by Raschmanová et al. The authors used single cell approaches to identify four sub-populations that varied in the unfolded protein response and other cellular parameters. Understanding and controlling this has important implications for efficient production of recombinant proteins.

Mercury is a potent antimicrobial but resistance mechanisms have evolved in many microbial species. The mercury resistance gene cluster (*mer*) of the mercury resistant marine bacterium *Pseudomonas stutzeri* was characterized by Zheng et al. The cluster encodes transcriptional regulators (MerR and MerD), the structural genes required for the transport (MerP and MerT) and reductive detoxification of Hg^2+^ to Hg^0^ (MerA) as well as MerF. The latter, which has homologs in several other pathogenic bacterial species, was shown by the authors to influence motility and biofilm formation and contribute to mercury resistance.

## Regulation of Transcription Under Stress

Dostálová et al. investigated the σ^D^ regulon of *Corynebacterium glutamicum*. The σ^D^ factor of RNAP, which belongs to a group of extracytoplasmic σ factors, was recently found to be involved in envelope stress response, synthesis of mycomembrane and formation of cell wall (Toyoda and Inui, 2018). The authors showed that σ^D^ increased the expression of 29 genes organized in 23 operons. Eleven promoter regions (encompassing 50 nucleotides) could be aligned and a consensus sequence derived. Using a combination of *in vitro* and *in silico* methods they provided evidence for overlapping functions of different sigma factors which should play a significant role in fine tuning of gene expression and coping with complex environmental stresses.

An interesting study on the global and specific changes in the m^5^C methylome was presented in the cyanobacterium *Synechocystis* sp. PCC 6803 by Hu et al. The authors demonstrated that 72 h of nitrogen starvation had no effect on the proportion of global m^5^C, rather on its distribution. In other words, nitrogen deficiency led to decrease of methylated sites, but the level of methylation of the m^5^C sites increased to an extent that balanced the decrease in the total methylated sites. The epigenetic pattern was partly inherited and still detectable after 12 generations, thus pointing to the methylome as a way to detect the stress history of a specific microorganism. Notably, no correlation was identified between level of methylation and increase/decrease of gene expression. Nitrogen stress was also investigated in the cyanobacterium *Nostoc* sp. PCC7120 by Álvarez-Escribano et al., though at the transcriptional and post-transcriptional level. The authors performed a detailed molecular study and demonstrated that a feed-forward loop is taking place involving the sRNA NsrR1 and the transcriptional regulator NtcA, which activated the gene *nblA*, encoding a protein adaptor for phycobilisome degradation under nitrogen deficiency. The authors showed that NtcA directly represses NsrR1, which in turn, when expressed, was responsible for repressing *nblA* expression by direct binding to its mRNA 5′-UTR thereby interfering with the translation start, possibly causing *nlbA* mRNA destabilization.

The iron starvation response is important for virulence in pathogenic yeasts, so Benchouaia et al. used a transcriptomic and bioinformatic approach to study this in the human pathogen *C. glabrata*. By focusing on genes that responded differently to iron stress in *C. glabrata* and other yeasts, they identified novel genes required for surviving iron starvation in *C. glabrata*. The REGULOUT bioinformatic tool described in this paper is likely to be applicable also for other comparative transcriptomics studies.

The importance of cyclic dinucleotides in stress signaling and in modulating adaptive gene expression was reflected in three different papers and three distinct bacteria. While c-diAMP has been implicated as a second messenger in cell wall homeostasis in response to osmotic stress in several bacterial species its role in the physiology of cyanobacteria had not been investigated. Agostoni et al. showed that c-di-AMP levels were modulated differently in two cyanobacterial species in response to osmotic stress (by salt or sorbitol), suggesting a role in osmotic stress adaptation in the genus *Synechocystis*. In *E. coli*, c-di-GMP is well-known to influence biofilm formation as a result of its role in regulating the production of surface structures called curli. Somorin et al. reported that some soil persistent strains of *E. coli* have lost curli production because of mutations that negatively affect c-di-GMP pools. These strains had a reduced capacity to produce biofilm, suggesting that there may be niches in the soil occupied by *E. coli* that do not require biofilm formation. The links between c-di-GMP and biofilm formation in the opportunistic human pathogen *P. aeruginosa* were investigated by Strempel et al. In this case, hypochlorite stimulated biofilm formation by inducing expression of a c-di-GMP synthase. Given that HClO is a common disinfectant, the potential to modulate the very complex intracellular c-di-GMP signaling network in a serious pathogenic bacterium highlighted the need to further explore and better understand such finely balanced systems.

## Author Contributions

All authors listed have made a substantial, direct and intellectual contribution to the work, and approved it for publication.

## Conflict of Interest

The authors declare that the research was conducted in the absence of any commercial or financial relationships that could be construed as a potential conflict of interest.
